# The bidirectional association between oral cancer and esophageal cancer: A population-based study in Taiwan over a 28-year period

**DOI:** 10.18632/oncotarget.17818

**Published:** 2017-05-11

**Authors:** Kuan-Der Lee, Ting-Yao Wang, Chang-Hsien Lu, Cih-En Huang, Min-Chi Chen

**Affiliations:** ^1^ Division of Hematology and Oncology, Department of Internal Medicine, Taipei Medical University Hospital, Taipei, Taiwan; ^2^ School of Medicine, College of Medicine, Taipei Medical University, Taipei, Taiwan; ^3^ Department of Hematology and Oncology, Chang Gung Memorial Hospital, Chiayi, Taiwan; ^4^ Department of Public Health and Biostatistics Consulting Center, College of Medicine, Chang Gung University, Taoyuan City, Taiwan

**Keywords:** oral cancer, esophageal cancer, second cancer, bidirectional association

## Abstract

Previous studies have revealed that patients with oral or esophageal cancer are at higher risk for subsequently developing a second primary malignancy. However, it remains to be determined what association exists between oral cancer and esophageal cancer particularly in Asian countries where squamous cell carcinoma is the predominant type of esophageal cancer. A population-based study was carried out in Taiwan, where the incidence rates of both oral and esophageal squamous cell carcinomas are high, to test the hypothesis that oral cancer or esophageal cancer predisposes an individual to developing the other form of cancer. Our results showed that patients with primary oral cancer (*n*=45,859) had ten times the risk of second esophageal cancer compared to the general population. Within the same cohort, the reciprocal risk of oral cancer as a second primary in primary esophageal cancer patients (*n*=16,658) was also increased seven-fold. The bidirectional relationship suggests common risk factors between these two cancers. The present study is not only the first population-based study in Asia to validate the reciprocal relationship between oral and esophageal squamous cell carcinomas, but also will aid in the appropriate selection of high-risk patients for a future follow-up surveillance program.

## INTRODUCTION

Oral cancer (including carcinoma of oral cavity, oropharynx and hypopharynx) and esophageal cancer are both prevalent cancers in Taiwan and their incidence rates rank fourth and seventh in cancer among males, respectively. It has been known that drinking and smoking are major risk factors in these two cancers [1–3]. Betel quid chewing is also related to a high incidence of oral and esophgeal cancers in Taiwan [4, 5], and the synergistic effects of cigarette, alcohol and betel quid use on the carcinogenesis of oral and esophageal cancers have been reported [6, 7]. Oral cancer patients are known to have a higher risk of second esophageal cancer than general population [8–10]. Due to short survival time of the esophageal cancer patients, the incidence of second primary malignancy after an index esophageal cancer is relatively small, and only a few reports have shown that patients with primary esophageal cancer are also at increased risk of developing a second oral cancer [11, 12]. Based on these observations, there is growing interest in understanding the bidirectional relationship between oral and esophageal squamous cell carcinomas.

A bidirectional association implies a reciprocal or two-way relationship between two different cancers, regardless of which one occurs first [13]. By contrast, a unidirectional association is defined as a one-way relationship between two cancer types; that is, having a primary cancer increases the risk of a subsequent cancer, but the reverse may not exist [13]. A bidirectional relationship between oral and esophageal cancers was first shown by Chuang et al [12]. In that study, they analyzed the dataset pooled from 13 cancer registries in Singapore, Australia, Canada and Europe. The results could be confounded by geographical and ethnic heterogeneity because only 4.9% cases were in Asia. In contrast to the Western countries that adenocarcinoma is the major histological type, more than 90% of the esophageal cancers in Asia are squamous cell carcinoma [14]. Thus, there is a clear need for another large-scale, single cohort study for Asian patients to add validity to the hypothesis that oral cancer or esophageal cancer predisposes an individual to developing the other cancer reciprocally. To achieve this goal, a population-based study was carried out by examining these two primary cancers and their risks of subsequent second malignancies using the same cohort population between 1979 and 2006 from the Taiwan Cancer Registry. The elucidation of the bidirectional relationship between two cancers will have important implications not only in understanding the etiologic mechanisms but also in early detection and treatment.

## RESULTS

### Patient characteristics

Among the patients with primary oral cancer (45,859 cases), including the oral cavity (35,854 cases), oropharynx (4,310 cases) and hypopharynx (5,695 cases), 3,611 cases (7.87%) developed at least one second primary malignancy (SPM) during 159,743 person-years of follow-up (Table 1). Of these 3,611 SPM cases, 357 cases (9.89%) were second esophageal cancer, and their mean age was 53.52 years at diagnosis of primary oral cancer and 56.26 years at diagnosis of the second cancer in esophagus, with an average interval of 2.74 years.

**Table 1:** Characteristics of the population-based cohort of 45,859 patients with a primary diagnosis of oral cancer (including the oral cavity, oropharynx, or hypopharynx), and 16,658 patients with a primary diagnosis of esophageal cancer, 1979-2006

Primary cancer site		Oral cancer				Esophagus
			**Oral cavity**	**Oropharynx**	**Hypopharynx**	
**(ICD-9 code)**			**(140–141, 143–145)**	**(146)**	**(148)**	**(150)**
No. with primary cancer	AllMF	45,85941,2514,608	35,85432,1603,694	4,3103,583727	5,6955,508187	16,65815,3091,349
Average (± SD) age at diagnosis of the first cancer (yrs.)		53.56 ± 12.57	52.75 ± 12.50	54.52 ± 12.78	57.92 ± 11.90	61.90±12.08
No. who developed a second primary cancer (%)*	AllMF	3,611 (7.87)3,332 (8.08)279 (6.05)	2,753 (7.68)2,533 (7.88)220 (5.96)	320 (7.42)278 (7.76)42 (5.78)	538 (9.45)521 (9.46)17 (9.10)	459 (2.76)434 (2.83)25 (1.85)
No. who developed the second esophageal cancer (%)*	AllMF	357 (0.78)352 (0.85)5 (0.11)	192 (0.54)191 (0.59)1 (0.03)	45 (1.04)44 (1.23)1 (0.14)	120 (2.11)117 (2.12)3 (1.60)	NA
Average (± SD) age at first diagnosis of the primary oral cancer for those who develop second esophageal cancer (yrs.)		(*n*=357)53.52 ± 9.91	(*n* =192)53.63 ± 10.10	(*n* =45)52.42 ± 9.99	(*n* =120)53.74 ± 9.62	NA
Average (± SD) age at diagnosis of the second esophageal cancer (yrs.)		56.26 ± 10.22	56.66 ± 10.36	53.69 ± 10.15	56.60 ± 9.96	NA
No. who developed second oral cancer (%)*	AllOral CavityOropharynxHypopharynx	NA	NA	NA	NA	132 (0.79)52 (0.31)26 (0.16)54 (0.32)
Average (± SD) age at first diagnosis of the primary esophageal cancer for those who develop secondary oral cancer (yrs.) (*n*=132)	AllOral CavityOropharynxHypopharynx	NA	NA	NA	NA	53.43 ± 10.0052.35 ± 9.8852.69 ± 9.3954.83±10.40
Average (± SD) age at diagnosis of the second oral cancer (yrs.)(*n*=132)	AllOral CavityOropharynxHypopharynx	NA	NA	NA	NA	55.99±10.4855.13±10.3155.04±9.6557.28±11.06
Average follow-up (yrs.)		3.48 ± 4.07	3.67 ± 4.10	3.33 ± 4.24	2.45 ± 3.52	1.98±3.64

*percentage of those with first primary cancer; yrs.=years.

Within the same cohort, the SPM after primary esophageal cancer was calculated. Among the patients with primary esophageal cancer (16,658 cases), 459 cases (2.76%) developed at least one SPM. Of these 459 SPM cases, 132 cases (28.76%) were second oral cancers (52 oral cavity, 26 oropharynx and 54 hypopharynx) and their mean age was 53.43 years upon diagnosis of primary esophageal cancer and 55.99 years upon diagnosis of second oral cancer, with an average interval of 2.56 years. Overall, there were 36,518 cases (58%) followed up for at least one year, 8,007 cases (13%) for 5-10 years and 4,694 cases (8%) for >10 years, and the mean follow-up time was 3.08 years.

### Risk of second cancer stratified by the anatomic site, follow-up time and age at diagnosis of primary cancer

The risk of second esophageal cancer was analysed in patients with primary oral cancer and *vice versa*. Standardized incidence ratios (SIRs) and corresponding 95% confidence intervals (CIs) were calculated by the anatomic site of oral cancer (Table 2). Among the patients with primary oral cancer, the risk of second esophageal cancer was increased (SIR=10.40, 95% CI= 9.35-11.53) and the risk was prominent as the primary index tumor was located in proximity to the esophagus: hypopharynx (SIR=29.28, 95% CI 24.27-35.01) > oropharynx (SIR= 16.04, 95% CI= 11.70-21.46) > oral cavity (SIR= 7.00, 95% CI= 6.04-8.06). Similarly, for the patients with primary esophageal cancer, the risk of second oral cancer was increased (SIR= 7.31, 95% CI= 6.11-8.67). The risk of second cancer in the anatomic site was also higher in proximity to the esophagus: hypopharynx (SIR= 16.72, 95% CI= 12.57-21.83) > oropharynx (SIR= 14.69, 95% CI= 9.62-21.58) > oral cavity (SIR= 3.98, 95% CI= 2.97-5.22).

**Table 2:** Risk of esophageal cancer as the second cancer site among 45,859 cases of primary oral cancer and the risk of oral cancer as the second cancer site among 16,658 primary esophageal cancer patients, 1979-2006

	**Primary oral cancer**			
	**All**	**Oral cavity**	**Oropharynx**	**Hypopharynx**
**Second esophagus**	**10.40**9.35–11.53357/34.34	**7.00**6.04–8.06192/27.44	**16.04**11.70–21.4645/2.81	**29.28**24.27–35.01120/4.10
	**Second oral cancer**			
	**All**	**Oral cavity**	**Oropharynx**	**Hypopharynx**
**Primary esophagus**	**7.31**6.11–8.67132/18.06	**3.98**2.97–5.2252/13.07	**14.69**9.62–21.5826/1.77	**16.72**12.57–21.8354/3.23

Three entries in each cell are SIR, 95% CI for SIR and O/E, where O = observed number of second primary cancers; E = expected number of second primary cancers.

Bold SIR indicates statistical significance.

The SIRs were stratified by time interval after the first diagnosis of primary cancer for exploring the latency of development of the second cancer. The follow-up time was divided into three categories: ≤5 years, 5-10 years and >10 years. For primary oral cancer patients, the risk of second esophageal cancer was the most predominant in the first 5 years, in which the sequence was hypopharynx (SIR= 53.33, 95% CI= 43.09-64.96) > oropharynx (SIR= 40.00, 95% CI= 28.94-53.47) > oral cavity (SIR= 16.14, 95% CI= 13.66-18.96), and decreased with longer follow-up but remained elevated for 10 years after diagnosis of the primary oral cancer (Table 3.1). For patients with primary esophageal cancer, a similar trend was found for the increased risk of second oral cancer in the first 10 years of follow-up time (Table 3.2).

**Table 3.1:** Risk of second esophageal cancer by follow-up interval after the first diagnosis of primary oral cancer

Primary cancer site	Follow-up time (yrs.)	SIR	95% CI	O/E
Oral cavity	≤5	**16.14**	13.66–18.96	149/9.22
	5–10	**4.43**	3.18–6.01	41/9.25
	>10	**0.22**	0.03–0.81	2/8.96
Oropharynx	≤5	**40.00**	28.94–53.47	44/1.10
	5–10	1.33	0.02–7.45	1/0.75
	>10	0	NA	0/0.95
Hypopharynx	≤5	**53.33**	43.09–64.96	96/1.80
	5–10	**17.89**	10.44–28.72	17/0.95
	>10	**5.19**	2.08–10.72	7/1.35

**Table 3.2:** Risk of second oral cancer by follow-up interval after the first diagnosis of primary esophageal cancer

Second cancer site	Follow-up interval (yrs.)	SIR	95% CI	O/E
Oral cavity	≤5	**5.98**	4.29–8.11	41/6.86
	5–10	**4.46**	2.14–8.21	10/2.24
	>10	0.25	0.00–1.40	1/3.97
Oropharynx	≤5	**26.67**	17.02–39.53	24/0.90
	5–10	3.33	0.04–18.72	1/0.30
	>10	1.79	0.02–9.85	1/0.56
Hypopharynx	≤5	**30.77**	22.75–40.91	48/1.56
	5–10	**7.55**	2.04–19.44	4/0.53
	>10	1.74	0.20–6.30	2/1.15

O = observed number of second primary cancers; E = expected number of second primary cancers.

Bold SIR indicates statistical significance.

In order to study where there is an age trend of second cancer, the SIRs were stratified according to three age groups (≤50, 50-60 and >60) at initial diagnosis of the primary cancer. For primary oral cancer, the risk of second esophageal cancer was high in younger patients, particularly those diagnosed before 50 years of age, with the sequence of hypopharynx (SIR= 73.77, 95% CI= 54.15-99.34) > oropharynx (SIR= 29.69, 95% CI= 17.79-46.18) > oral cavity (SIR= 11.34, 95% CI= 9.10-13.98) (Table 4.1). Similarly, in patients with primary esophageal cancer, the risk of second oral cancer was the highest in those aged ≤50 (hypopharynx SIR= 56.41, 95% CI= 35.67-86.20; oropharynx SIR= 36.67, 95% CI= 18.14-65.10; oral cavity SIR= 11.02, 95% CI= 7.31-15.91) (Table 4.2).

**Table 4.1:** Risk of second esophageal cancer by age at initial onset among 45,859 patients with primary oral cancer

Primary cancer site	Age at diagnosis of primary oral cancer (yrs.)	SIR	95% CI	O/E
Oral cavity	≤ 50	**11.34**	9.10–13.98	88/7.76
	50–60	**5.65**	4.22–7.40	52/9.21
	> 60	**4.97**	3.71–6.51	52/10.47
Oropharynx	≤ 50	**29.69**	17.79–46.18	19/0.64
	50–60	**19.78**	11.78–31.44	18/0.91
	> 60	**6.35**	2.74–12.53	8/1.26
Hypopharynx	≤ 50	**73.77**	54.15–99.34	45/0.61
	50–60	**35.71**	26.05–47.80	45/1.26
	> 60	**13.45**	9.06–19.18	30/2.23

**Table 4.2:** Risk of second oral cancer by age at initial onset among 16,658 patients with primary esophageal cancer

Second cancer site	Age at diagnosis of primary esophageal cancer (yrs.)	SIR	95% CI	O/E
Oral cavity	≤50	**11.02**	7.31–15.91	28/2.54
	50–60	**3.17**	1.73- 5.32	14/4.41
	> 60	1.64	0.78–3.01	10/6.11
Oropharynx	≤50	**36.67**	18.14–65.10	11/0.30
	50–60	**18.18**	8.63–33.15	10/0.55
	>60	**5.49**	1.77–12.85	5/0.91
Hypopharynx	≤50	**56.41**	35.67–86.20	22/0.39
	50–60	**17.71**	10.36–28.49	17/0.96
	>60	**7.94**	4.45–13.12	15/1.89

O = observed number of second primary cancers; E = expected number of second primary cancers.

Bold indicates statistical significance.

### Cumulative incidence of second cancer

**Figure 1:**
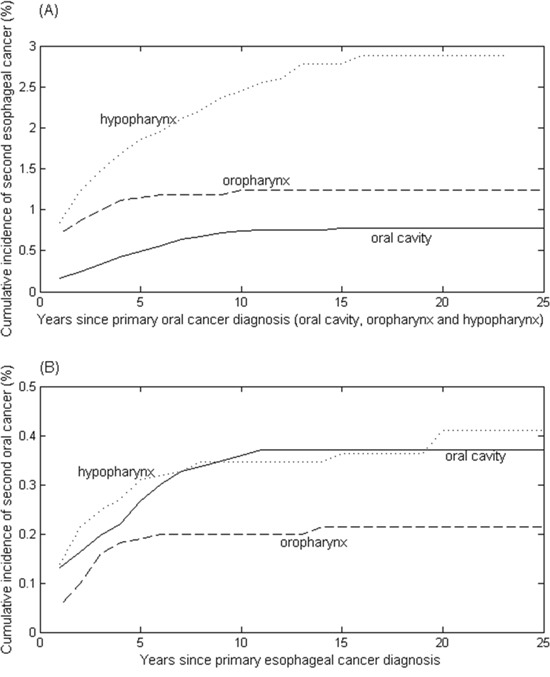
Cumulative incidence rates of **(A)** second esophageal cancer in a total of 45,859 patients with primary oral cancer, including the oral cavity (35,854 cases), oropharynx (4,310 cases) and hypopharynx (5,695 cases); and **(B)** second oral cancer in a total of 16,658 patients with primary esophageal cancer.

The cumulative risk of developing second esophageal cancer in oral cancer survivors was estimated by treating death and non-esophageal cancers as competing risks (Figure 1A). The highest overall cumulative incidence of second esophageal cancer was observed in the patients with primary hypopharynx, followed by oropharynx and oral cavity. There was a significant difference in the incidence of esophageal cancer among three oral cancer groups (all *P*-values <0.01).

Similarly, the cumulative probability of developing second oral cancer after primary esophageal cancer was estimated (Figure 1B). The cumulative risk of second oral cavity was not different from that of second hypopharynx (*P*-value = 0.845), revealing that the patients with primary esophageal cancer were at similar risk for developing second oral cavity and hypopharyngeal cancers. In contrast, the cumulative incidence of developing second oropharyngeal cancer was much lower than that of developing second oral cavity and hypopharynx (*P*< 0.02).

### Overall survival of the primary oral cancer or esophageal cancer patients

**Figure 2:**
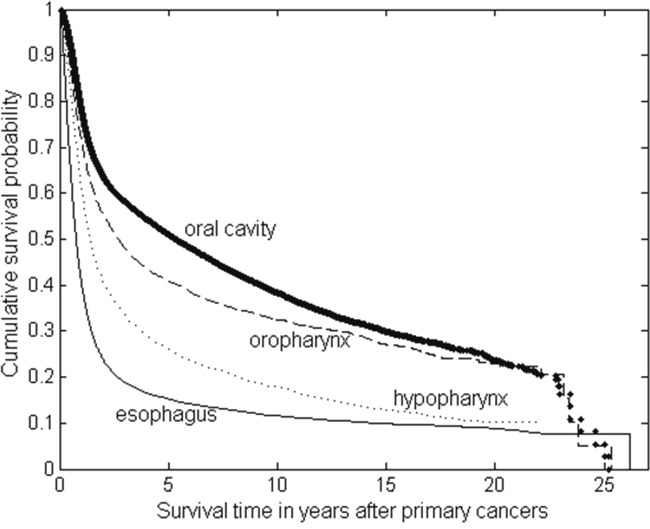
The Kaplan-Meier survival curves of the patients with primary oral cancer (including oral cavity, oropharynx and hypopharynx) and primary esophageal cancer

With the overall survival defined as the time from the date of primary cancer diagnosis to death from any cause, the median survival time and 5-year survival rate for all oral cancer patients were 4.06 years and 46.90%. For stratification by the primary tumour site, the median survival times were 5.32, 2.70, 1.42, 0.76 years for the oral cavity, oropharynx, hypopharynx and esophagus, respectively. The survivals were significantly different from each other (all *P*-values < 0.001), in which survival was highest in patients with oral cavity (5-year survival rate: 51%) and lowest in those with esophagus (5-year survival rate: 15.3%) (Figure 2).

### Survival time after second cancer occurrence

**Figure 3:**
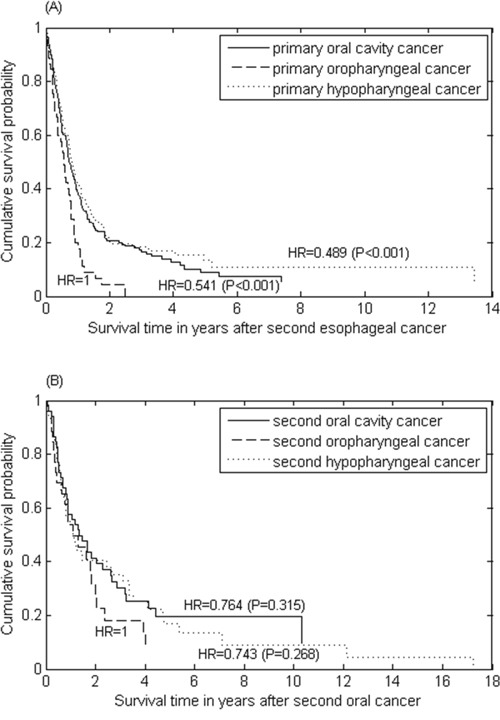
The survival curves of **(A)** second esophageal cancer for patients with primary oral cancer stratified by index tumour site; and **(B)** second oral cancer for patients with primary esophageal cancer.

The survival time after occurrence of second cancer was poor for the patients with either primary oral cancer or primary esophageal cancer. The survival curves were compared by adjusted hazard ratios from a Cox proportional hazards model. For primary oral cancer patients who had second esophageal cancer, the median survival after second cancer was only 0.73, 0.57, and 0.81 years for the primary oral cavity, oropharynx and hypopharynx, respectively, in which the oral cavity and hypopharynx had better survival compared to the oropharynx (HR=0.54 and HR=0.49 with *P*-values < 0.001) (Figure 3A). For primary esophageal cancer patients who had a second oral cancer, the survival was also poor regardless of the anatomic site of second oral cancer (*P*-values > 0.27) (Figure 3B).

## DISCUSSION

In our cohort, patients with primary oral cancer had an increased risk of second esophageal cancer (SIR=10.40, 95% CI= 9.35-11.53). The risk was increased as the primary tumor site was in the proximity of the esophagus, most frequently seen in hypopharyngeal cancer, followed by oropharyngeal cancer and the oral cavity cancer. Within the same cohort, the reciprocal risk of second oral cancer in patients with primary esophageal cancer was also increased (SIR= 7.31, 95% CI= 6.11-8.67), and the frequency of second cancer location was seen in a similar sequence: hypopharynx> oropharynx> oral cavity. This bidirectional relationship suggests common risk factors between oral cancer and esophageal cancer. Although the real pathogenesis has not yet been clarified, several mechanisms seem plausible. First, field cancerization [15] can partially explain the development of multiple tumours, especially in survivors of certain cancers who are prone to develop other malignancies of the same tissue type near the primary cancer. In Taiwan, 57% of adult males smoke cigarette, 46% drink alcohol, and 15% chew betel quid [16]. The whole epithelial lining of the oral and upper digestive tract is commonly exposed to these three carcinogenic factors and the resulting increase in cancer risk. The epigenetic alterations of some genes, such as *HOXA9*, have been implicated in both oral cancer and esophageal cancer patients as potential biomarkers for the early detection of the field of cancerization [17–19]. The second possible mechanism is the shared genetic alterations for carcinogenesis. There is increasing evidence that younger cancer patients are more likely to have genetic predispositions. In line with this, our analysis showed that the risk of either oral cancer or esophageal cancer as a second cancer was both extraordinarily high for patients with initial cancer diagnosed at age ≤50. These genetic alterations may give rise to the dysregulation of cell proliferation and differentiation pathways. For example, the tumour suppressor gene *TP53* plays an important role in the multi-step carcinogenesis for both cancers, and *TP53* mutations were found in 93% of esophageal squamous cell carcinoma [20] and 57% of oral squamous cell carcinoma in Chinese patients, particularly those who had alcohol drinking, betel quid chewing or cigarette smoking [21]. Recently, genetic polymorphisms of acetaldehyde dehydrogenases (ALDH2) gene is recognized as a key factor regarding the susceptibility to both esophageal and oral squamous cell carcinomas. The ethanol in alcohol is metabolized to acetaldehyde, and the acetaldehyde is metabolized to acetate by acetaldehyde dehydrogenases in the liver. Inactive heterozygous ALDH2 alleles cause a deficiency of the enzyme and were shown to increase the risk of esophageal squamous cell carcinoma (SCC) and metachronous head and neck cancer. In Taiwan and Japan, 65%–76% of esophageal cancer patients carried the ALDH2 risk alleles [22–25].

Another possible shared aetiology is human papilloma virus (HPV), as either a direct carcinogen or promoter, in oral and esophageal carcinomas. In addition to cervical cancer, HPV has been found to be strongly associated with head and neck cancer [26], particularly oropharyngeal squamous cell carcinoma, where it is detected in 40-60% of patients [27]. As HPV DNA has also been confirmed in esophageal cancer patients, it is plausible that HPV can infect the squamous epithelium of the esophagus in the same manner as the oropharynx [28–30]. On top of that, the *in vitro* and animal studies showed that HPV16 E6-E7 can induce cancer stem-like cells phenotypes in esophageal squamous cell carcinoma through activation of the PI3K/Akt signalling pathway [31]. However, a geographical difference was found in the proportion of HPV DNA in esophageal cancer tissues, which ranges from 6% to 65% [32, 33], suggesting that the risk factors for esophageal cancer could be heterogeneous and more studies are warranted to evaluate the possible aetiological role of HPV in esophageal cancer.

Patients who survive longer have a longer risk period in which a second primary cancer may develop. In this analysis, the survival of primary esophageal cancer was much shorter than that of oral cancer (5-year survival 15.3±0.29% *versus* 46.90±0.20%, and median survival 0.76±0.01 *versus* 4.06±0.07 years, respectively). Thus, we found the incidence of all SPMs after an index esophageal cancer was smaller than after an index oral cancer (2.76% *versus* 7.87%). Of these SPMs, intriguingly, the incidence of second oral cancer following an index esophageal cancer is similar to the incidence of second esophageal cancer after an index oral cancer (0.79% *versus* 0.78%). This is probably because the SIRs for second oral or second esophageal cancer following a first primary cancer are the highest during the first year of follow-up. However, the highest excess risk seen in the first year of follow-up might be biased by close surveillance or misclassification. Regardless of the primary site, the median survival for second esophageal or oral cancer was less than one year. The dismal prognosis may be in part due to the difficulties in receiving aggressive therapy.

In contrast to the U.S., squamous cell carcinoma (SCC) accounts for 95% of all esophageal cancers in Taiwan. Although most countries including Taiwan follow the National Comprehensive Cancer Network (NCCN) guideline which did not recommend routine upper gastrointestinal endoscopy for oral cancer patients or regular oral examination by otolaryngologists for esophageal cancer patients for follow-up. However, surveillance endoscopy has been highly recommended for high risk patients with oral cancers in Taiwan [34]. Wang et al reported that esophageal squamous cell carcinomas and high-grade intraepithelial neoplasms were found in 10.1% and 7.3%, respectively, of the 441 patients with head and neck cancers receiving the endoscopy screening [35]. Based on our present study enlightening the reciprocal relationship between oral and esophageal cancers by their risk of developing a second cancer, it cannot be over-emphasized that both oral examination by otolaryngologists and endoscopy should be performed in the screening and surveillance program.

## MATERIALS AND METHODS

### Data sources

We quantified the risk of second cancer incidence among patients who were recorded with the diagnosis of oral cancer or esophageal cancer to the Taiwan Cancer Registry (TCR) (http://tcr.cph.ntu.edu.tw/) between 1 January, 1979 and 31 December, 2006. The TCR was founded in 1979 and is financed by the Ministry of Health and Welfare for estimating the incidence of cancer in Taiwan. It is a population-based cancer registry that covered 22 million people and 97.6% of cancer patients in 2006 [36]. All cancer registry databases in the TCR have been systemically converted to International Classification of Diseases, 9th Revision codes [37], and linked with death certificates last updated in December, 2007 from the National Death Database. Persons not identified by this process were therefore considered to be alive for the purposes of the current study (passive follow-up). The coding of multiple primaries followed the principles of International Association of Cancer Registries (IACR) and the International Agency for Research on Cancer (IARC) [38, 39]. Informed consent was not required because all registry records are anonymous and accessible to the public.

To assess the age of onset, estimate the person-year follow-up and minimize the potentially unconfirmed cancer diagnosis in this study cohort, 5,673 patients were excluded from analysis because they met one or more of the following criteria: (1) missing birth dates or unknown gender (20 cases), (2) missing last follow-up date or death status (1,234 cases), (3) the follow-up time (310 cases), second cancer diagnosis (2,377 cases) or death (3,355 cases) occurring less than 1 month after the primary cancer, or (4) age under 20 years old (138 cases). As a result, a total of 62,517 cases (56,560 males and 5,957 females) were included in the analysis. Of them, 45,859 patients had primary oral cancer, which includes the primary site of the oral cavity (ICD-9:140-145 except 142), oropharynx (including the soft palate, tongue base and tonsil; ICD-9: 146) and hypopharynx (including hypopharynx and pyriform sinus; ICD-9: 148), and 16,658 patients had primary esophageal cancer (ICD-9: 150).

### Statistical analysis

To quantify the excess of second malignancies after the diagnosis of primary oral cancer or esophageal cancer, we calculated the standardized incidence ratios (SIRs) [40], and the corresponding 95% confidence intervals (CIs) for some specific types of second primary cancers. SIRs were taken as the ratio of the observed number (O) of second cancers to the expected number (E), which was obtained by assuming that these persons experienced the same cancer incidence as the corresponding general population. The number of person-years at risk was defined as the number of years from the date of initial diagnosis of the primary cancer to the date of death, date of last follow-up, date of the diagnosis of second primary cancer, or the end of the study period (31 December, 2006), whichever came first. The person-years of observation for each gender, 5-year age group and 5- or 3-year period (1979-1983, 1984-1988, 1989-1993, 1994-1998, 1999-2003, 2004-2006) were multiplied by the incidence rates of cancers for the Taiwanese population. The corresponding products were summed over all ages, genders and calendar years to yield the expected number of second cancers at each site. Confidence intervals of SIRs were based on the assumption of a Poisson distribution of second cancer cases.

Cumulative incidence rates for the occurrence of second cancers were calculated in the survivors’ cohort, with death treated as a competing risk according to the method of Kalbfleisch and Prentice [41]. Briefly, this method allows for the fact that patients who die are no longer at risk for second cancers, so it differs from the cumulative incidence estimated by the Kaplan-Meier method, which treats competing events as censored at the time they occurred. Gray's test was used to assess the significant differences of the cumulative incidence between two primary index tumours [42]. The Kaplan-Meier survival curves were used to present the survival time after primary cancers and after second cancers. The overall survival was compared by log rank test, and the differences among curves after second cancers were presented by hazard ratio using the Cox proportional hazards model, in which gender and age at onset were adjusted. All statistical tests were two-sided and *P* <0.05 was considered statistically significant.

## Abbreviations

CI: confidence interval
HPV: human papilloma virus
IACR: International Association of Cancer Registries
IARC: International Agency for Research on Cancer
NA: not applicable
SD: standard deviation
SIR: standardized incidence ratio
SPM: second primary malignancy
TCR: Taiwan Cancer Registry
